# An educational video to promote the use of stigma-free language by primary care clinicians in interactions with adults with type 2 diabetes: a qualitative study

**DOI:** 10.1017/S1463423625100728

**Published:** 2025-12-29

**Authors:** Kevin Joiner, Alexandra Agapiou, Jane K. Dickinson, Mackenzie Adams, Gretchen Piatt

**Affiliations:** 1 Department of Health Behavior and Clinical Sciences, School of Nursing, University of Michigan, Ann Arbor, MI, USA; 2 Department of Systems, Populations and Leadership, School of Nursing, University of Michigan, Ann Arbor, MI, USA; 3 Department of Health & Behavior Studies, Teachers College, Columbia University, New York, NY, USA; 4 Department of Learning Health Sciences, Medical School, University of Michigan, Ann Arbor, MI, USA; 5 School of Public Health, University of Michigan, Ann Arbor, MI, USA

**Keywords:** diabetes, primary care, stigma

## Abstract

**Aim:**

The aim of this study was to explore the acceptability of an educational video among primary care clinicians as a tool to promote the use of stigma-free language in interactions with individuals with type 2 diabetes (T2D).

**Background:**

The language used by primary care clinicians in interactions with adults living with T2D can contribute to perceptions and experiences of diabetes-related stigma and be a barrier to achieving and sustaining glycaemic targets. In 2017, the American Diabetes Association (ADA) and the Association for Diabetes Care & Education Specialists (ADCES) issued a guidance paper with recommendations to promote stigma-free communication about diabetes.

**Methods:**

The educational video, developed by the research team, presents two versions of a vignette in which a nurse practitioner interacts with an adult with T2D in a primary care setting. The first version of the vignette features the nurse practitioner using stigmatizing language as outlined in the ADA and ADCES guidance paper; the second demonstrates the use of stigma-free language by the nurse practitioner. A narrator highlights the linguistic differences. The study participants, comprising physicians (*n* = 8), nurse practitioners (*n* = 9), and physician assistants (*n* = 1), were recruited through professional networks and via online forums and listservs for healthcare professionals. Participants viewed the educational video and were interviewed via Zoom by a research team member using a semi-structured interview guide. The transcripts of the interviews were analysed using a qualitative descriptive approach.

**Findings:**

Three main themes emerged from the data: aligning video content with existing attitudes and beliefs, reducing the use of stigmatizing language, and increasing the use of stigma-free language. Findings suggest that an educational video promoting the use of stigma-free language in interactions with adults with T2D is acceptable among primary care clinicians.

## Introduction

Approximately 11.6 percent of adults in the U.S. are living with diabetes, 29.4 million adults have been diagnosed, and 8.7 million adults remain undiagnosed. (Centers for Disease Control and Prevention, 2024) Among U.S. adults with diabetes, the majority have type 2 diabetes (T2D). For adults with T2D, achieving and maintaining glycaemic levels that meet recommended targets, as well as numerous other factors, can help to ensure optimal health. (American Diabetes Association [ADA] Professional Practice Committee, 2025c) Fortunately, many adults with T2D have access to medical care and health care resources as well as social and educational support for diabetes self-management. (Edelman and Polonsky, 2017; Khunti *et al.*, 2018)

For adults with T2D to fully leverage available healthcare resources and address clinical needs, it is vital to maintain effective communication with primary care clinicians as they collaborate to find solutions to challenges. (Kelley *et al.*, 2014) Evidence suggests that primary care clinicians sometimes use language that communicates diabetes-related stigma. (Speight *et al.*, 2024; Park *et al.*, 2021) The use of stigmatizing language by primary care clinicians may contribute to perceptions and experiences of adults with T2D, including blame, (Browne *et al.*, 2013) judgement, (Dickinson *et al.*, 2023) stereotyping, rejection, exclusion, and discrimination. (Speight *et al.*, 2024) Internalization of such perceptions and experiences by adults with T2D can manifest as feelings of embarrassment and shame. (Browne *et al.*, 2013; Bennett and Puhl, 2024) Furthermore, diabetes-related stigma may contribute to the disconnect that sometimes exists between patients’ intentions to improve their health and primary care clinicians’ perceptions, which may incorrectly attribute treatment challenges to lower patient motivation. (Khunti *et al.*, 2018) By using stigmatizing language, primary care clinicians could be less effective in building and maintaining trust, which might result in adults with T2D avoiding conversations or support for emotional experiences and self-care challenges. (Dickinson *et al.*, 2023; Litterbach *et al.*, 2024)

In 2017, the American Diabetes Association (ADA) and the Association for Diabetes Care & Education Specialists (ADCES) issued recommendations for stigma-free discussions about diabetes, including a list of preferred terms. (Dickinson *et al.*, 2017) These recommendations have been incorporated into the ADA Standards of Care for Diabetes. (ADA Professional Practice Committee, 2025a; ADA Professional Practice Committee, 2025b) Despite the evidence of diabetes-related stigma and its effects, research specifically targeting approaches to address stigma remains limited. (Speight *et al.*, 2024; Eitel *et al.*, 2024) Educational videos have been successfully used in other healthcare contexts as tools to address stigma. (Talumaa *et al.*, 2022) The research team developed an educational video as a tool to promote the use of stigma-free language among primary care clinicians when interacting with adults with T2D. This study was designed to assess the acceptability of this educational video among primary care clinicians. Insights from this study will inform the further refinement and enhancement of the educational video for integration into an intervention and broader application.

## Setting

Primary care serves as the main point of contact for diabetes management in the U.S., with approximately 90% of adults with T2D receiving care in these settings. (Bannuru *et al.*, 2024) The national primary care workforce consists of approximately 50% physicians, 45% nurse practitioners, and 5% physician assistants, and care is commonly delivered by multidisciplinary teams, including Certified Diabetes Care & Education Specialists (CDCESs) and registered dietitians. (Health Resources & Services Administration [HRSA] National Center for Health Workforce Analysis, 2024; ADA Professional Practice Committee, 2025c) This study was conducted at the University of Michigan, located in Washtenaw County, Michigan. Michigan’s scope-of-practice laws permit nurse practitioners and physician assistants to initiate and adjust diabetes medications in collaboration with physicians. (National Conference of State Legislatures [NCSL], 2025a; NCSL, 2025b) Many regions in Michigan, including parts of Washtenaw County, are federally designated as primary care Health Professional Shortage Areas (HPSAs), presenting access challenges for many patients. (Rural Health Information Hub, 2025) While the study was conducted at the University of Michigan, participants were recruited from both Michigan and nationwide.

## Materials and methods

### Educational video

Based on ADA and ADCES recommendations, the research team developed an educational video designed to promote the use of stigma-free language among primary care clinicians when interacting with adults with T2D. The video presents two vignettes featuring a primary care nurse practitioner and an adult with T2D discussing self-care challenges, outcomes, and treatment options in an outpatient clinic setting. The first vignette features language that may be perceived or experienced as stigmatizing, such as referring to the adult with T2D as ‘a diabetic’ and ‘non-compliant’. The second vignette offers alternative phrasing, including ‘a person with diabetes’ and ‘not taking medication as prescribed’. Throughout the video, a narrator highlights these differences, guiding viewers to recognize stigmatizing language and alternative stigma-free language. The entire video lasts 11 minutes and 47 seconds.

### Study design

This study employed a qualitative descriptive approach to gather insights into the acceptability of the educational video among primary care clinicians. (Sandelowski, 2000) Semi-structured interviews were used to collect detailed perspectives, focusing on real-life contexts and previous experiences related to diabetes communication. (Sandelowski, 2000) Participants were recruited through professional networks and via professional forums and listservs targeting healthcare professionals. Eligibility criteria included being an actively practising primary care clinician (physician, nurse practitioner, or physician assistant) in the U.S. who routinely provides care for adults with T2D. There were no exclusion criteria.

Interested individuals completed an online screener and supplied their contact information. A member of the research team subsequently contacted these individuals via email or phone. Those who agreed to participate were scheduled for an online meeting via Zoom. Before the meeting, they were given access to the educational video and instructions on how to view it. During the online meeting, after obtaining verbal informed consent, the researcher asked the participant if they had viewed the educational video beforehand. If not, the video was shown to the participant during the online meeting, prior to conducting the semi-structured interview. The semi-structured interview guide was designed to explore participants’ perceptions of the acceptability of the educational video, specifically addressing factors influencing engagement and perceived relevance, perceived barriers and supports, and suggestions for improvement (see Table 1).

**Table 1.** Guide for semi-structured qualitative interviews

Question	Factor explored^a^
1. What is the most powerful message you gained from the video?	Engagement and perceived relevance
2. What are some examples from this video that you can relate to your work?	
3. What do you think about how realistically the actors portray the patient-provider communication encounter?	
4. What do you think about how the narrator spoke?	
5. What are your thoughts on how the video looks overall?	
6. What are the benefits of a diabetes-related stigma intervention?	Perceived barriers and supports
7. What did you learn that you can adopt into your own practice?	
8. Would you recommend this video to any other healthcare worker in your office (e.g., physician, physician assistant, patient care technician)?	Suggestions for improvement
9. What recommendations do you have for how we can improve the video?	
10. How do you think this video can induce change in your understanding and attitudes toward language use and diabetes-related stigma?	

a Interview questions were designed to explore factors influencing participants’ perceptions of the acceptability of the educational video.

The sociodemographic information collected included age, gender, race, and ethnicity. The professional practice information collected included clinician type, years of primary care experience, number of primary care appointments with patients with T2D in a typical day, credential status as a Certified Diabetes Care and Education Specialist (CDCES), practice location, and practice setting. Upon completing the semi-structured interview, participants were emailed a $40 gift card as compensation for their time and participation.

### Data analysis

The research team member who conducted the semi-structured interviews transcribed the recordings. The transcripts were reviewed and updated as needed for accuracy. The researcher and a second member of the research team then independently reviewed all the transcripts. Each created a list of potential codes with detailed descriptions. These two researchers, along with the research team leader, developed a codebook through consensus based on these lists. Using the computer-aided qualitative data analysis software, ATLAS.ti^TM^ (Scientific Software Development, Berlin, Germany), the two research team members then independently applied these codes to text segments. As new concepts emerged, additional codes were inductively added to the codebook. Following the coding, discrepancies were identified and discussed among the two research team members and the research team leader, and any differences were resolved through consensus. Computer-aided qualitative data analysis software was used to organize the textual data, identify patterns, and facilitate consensus on the final themes and subthemes to be reported. After the interviews were conducted, data analysis revealed consistent themes that demonstrated sufficient depth to address the study’s objectives.

### Ethical considerations

The study was approved as exempt by the Institutional Review Board of the University of Michigan’s Health Sciences and Behavioral Sciences (HUM00207526).

## Results

### Sample characteristics and identified themes

Eighteen interviews were conducted. One-third (*n* = 6) of the participants were 40 years or older, the majority (*n* = 14) identified as female, and most (*n* = 14) reported having non-Hispanic white race and ethnicity. The sample consisted of approximately half (*n* = 9) nurse practitioners, followed by physicians (*n* = 8), and a physician assistant (*n* = 1). On average, participants had 5.2 years of primary care experience. Most reported seeing between one and five adults with T2D in a typical day. The majority practiced in urban settings and in a group practice or a private office (Table 2).

**Table 2.** Demographic and practice characteristics of participants (*N* = 18)

Characteristic	n or mean (SD)
Age, years	
20–29	4
30–39	8
40+	6
Sex	
Female	14
Male	4
Race/Ethnicity	
Non-Hispanic White	14
Asian	4
Primary Care Clinician Type	
Physician	8
Nurse Practitioner	9
Physician Assistant	1
Primary Care Experience, years	5.2 (6.8)
Appointments with patients with type 2 diabetes per day	
1–5	11
6–10	6
>10	1
Certified Diabetes Care & Education Specialist	
Yes	1
No	17
Primary Care Practice Location	
Urban	11
Suburban	4
Rural	3
Primary Care Practice Setting	
Group Practice or Private Office	12
Federally Qualified Health Center	5
Home-based Managed Care Service	1

Three primary themes emerged from the qualitative data: aligning video content with existing attitudes and beliefs, reducing the use of stigmatizing language, and increasing the use of stigma-free language.

#### Aligning video content with existing attitudes and beliefs

For some participants, the educational video prompted them to reflect on their existing attitudes and beliefs regarding language use and its positive and negative impact on T2D care and outcomes. For instance, one participant recounted his journey from initially experiencing communication challenges in interactions with adults with T2D to enhancing his awareness and adjusting his approach over time. He reflected on earlier patient interactions:

I probably came off pretty abrasive the very first year. I would get angry when patients’ A1C [haemoglobin A1C] levels were over 9. As time goes on, you realize that you’re there to effect change, and you need to do that without becoming upset. Because that doesn’t do either of you any good; when you get angry with somebody or start telling them that they need to do this or need to do that, they just shut down (Participant 2, nurse practitioner, 6 years of experience).

Another participant noted an unintentional yet meaningful shift in their language use in the context of T2D, which had become a deliberate practice over time. ‘I will say “people with diabetes” instead of “diabetic.”. I didn’t realize I made that distinction. It was by accident, not on purpose. But now I feel much more cognizant that it’s actually a better way to do it’. (Participant 7, nurse practitioner, 25 years of experience). These insights, which some participants reported experiencing after viewing the educational video, highlight a potential effect on raising awareness of the value of using stigma-free language.

Several participants stated that the educational video reaffirmed their existing perspectives on the ethical imperative of employing respectful language in T2D care in laying the groundwork for positive clinician-patient relationships. One participant encapsulated this sentiment this way: ‘I feel the message is to remember that these are people, not just a disease, and to treat them with respect and be patient centered. Include them and make them feel like a person’. (Participant 4, nurse practitioner, 0.5 year of experience). Beyond ethical concerns, participants commented on the connection between the health professionals’ use of language in interactions with adults with T2D and the therapeutic efficacy of positive clinician-patient relationships. One participant articulated the broader implications:

It [the clinician’s use of language] helps build rapport, … making the patient feel safe to share what they’re going through. If the patient doesn’t feel safe, … they may hesitate to share issues like the financial burdens of their medications… A patient might say, ‘I’m taking metformin every single day, and I don’t have any side effects or financial issues.’ But that may not be true. (Participant 14, physician, 8 years of experience).

#### Reducing the use of stigmatizing language

A key theme identified in the data was the acknowledgement of difficulties in reducing the use of stigmatizing language in healthcare. Despite a commitment to being mindful of messages, some participants identified the challenges inherent in changing deeply ingrained habits. Factors such as time constraints, staffing shortages, long work hours, and provider burnout were discussed as barriers that impede a focus on stigma-free communication during patient interactions, particularly with individuals with T2D. One participant described: ‘The first thing that came to mind was how things can get so busy and hectic. There’s not a whole lot of time to reflect before or after appointments’ (Participant 9, physician, 1 year of experience). Another participant, commenting on the fast-paced nature of contemporary healthcare interactions, stated:

It’s simple but goes back to the basics that healthcare providers and nurse practitioners often forget. You have to think about what you say before you say it. Right now, the healthcare world is so different; everyone is overworked and short-staffed. People are rushing conversations and interactions, and I don’t think they take the time to think before they speak (Participant 6, nurse practitioner, 1.5 years of experience).

Some participants noted that, from their perspective, many of their colleagues demonstrated the use of stigma-free language in their conversations about T2D. Participants also shared their views that some colleagues seemed to harbour deep-seated beliefs that influenced their communication. As one participant explained:

There is a challenge in changing the culture of how providers think, starting from early medical school… Changing the culture of not saying shaming things behind patients’ backs is important so that this attitude doesn’t permeate the exam room to patients. Generally, people don’t say really rude or shaming things … but some undertones might come through (Participant 17, physician, 11 years of experience).

Some participants pointed out that they viewed a gap between the clinical terminology traditionally used in healthcare settings and the imperative of crafting their communication in a way that will help reduce the perpetuation of diabetes-related stigma. Some participants discussed how the educational video effectively illustrated clinician-patient communication, prompting their desire for further opportunities to reinforce the use of words and phrases free from stigma in both spoken and written interactions about T2D. One participant remarked:

The terminology in the medical space means something different to laypeople, so it’s difficult….I’ve tried using ‘non-adherent,’ but … I haven’t found a good word to describe the situation. The video showed the provider validating the patient by saying, ‘People miss pills, it’s okay, thanks for telling me.’ That’s a good way to react. … It would be helpful to include notes … in the training so trainees can see how this is implemented, not only in how you speak with patients, but how you write about them. (Participant 13, physician, 4 years of experience).

Concerns about the perpetuation of diabetes-related stigma were also discussed. Another participant highlighted: ‘Calling people ‘a diabetic’ is popular vernacular in healthcare. We always call people “a diabetic,” but we don’t call people “a hypertension” or “a heart attack”’ (Participant 5, nurse practitioner, 0.5 year of experience). Resistance from colleagues with traditional mindsets was noted, with one participant commenting on the dismissal of newer concepts:

Another barrier is that physicians or clinicians who are older or just have a more traditional mindset may not find these ideas useful and might scoff at them… What could be helpful … is saying ‘here’s why we don’t want to use the word “diabetic”, patients feel worse, and their mental health is worse’. That becomes much more convincing than saying, ‘Use this word because we think it feels nice’ (Participant 16, physician, 1 year of experience).

Moreover, stigmatizing language was perceived as indicative of broader systemic issues within healthcare, where power dynamics might amplify patient reluctance to express dissatisfaction or concerns. A participant commented:

It’s a paternalistic approach type of thing… People feel you have more power over them, which is inherent in the relationship… Meeting people on their level is really important. And not using words that reinforce the bad feelings they might already have about themselves is important. Because people have bad feelings about themselves, like, you know, ‘It’s just God’s judgment’, ‘I ate too many chocolate bars’, ‘I didn’t exercise enough’ (Participant 7, nurse practitioner, 25 years of experience).

#### Increasing the use of stigma-free language

A prominent theme that emerged from the data was the emphasis on the importance of planning for practice and anticipating progress in using stigma-free language in interactions with adults with T2D. The educational video prompted some participants to express a commitment to be more deliberate in their communication regarding T2D, expecting that sustained practice would foster meaningful improvements. One participant captured this idea, stating: ‘I think if you make it a habit to eliminate words like ‘diabetics,’ ‘poor control,’ and ‘non-compliance’ from your language, and practice that over and over again, it can change how you speak to patients’ (Participant 3, nurse practitioner, 0.5 year of experience). Participants also discussed incorporating scripting and practice tools into their therapeutic approaches. One noted the value of strategies:

I always find scripting helpful… At my desk, I have a list of words that I reference sometimes, not just about diabetes but about things in general, to sound neutral and non-judgmental. So, anytime someone can give me a script or ideas on how to phrase things, I am all about that (Participant 1, nurse practitioner, 0.5 year of experience).

Some primary care clinicians pointed out the convenience offered by the educational video’s format. One participant noted: ‘The good thing is the way … it [the video] allows them [clinicians] to watch it at their convenience, on their own time. As a busy primary care clinician myself, I think that’s really helpful’. (Participant 15, nurse practitioner, 18 years of experience). Some participants emphasized the utility of educational tools that incorporate aspects of role-play and self-awareness as a means for professional development. A participant remarked: ‘Videos and sample visits are super helpful because you can see yourself in the clinician’s shoes and identify, like, ‘Oh, I do that.’ Having a sample of what language to use and a video with that is really great’ (Participant 18, physician, 2.5 years of experience).

Participants expressed optimism about fostering change toward using stigma-free language in diabetes care and recognized the potential of integrating tools, such as educational videos, into career development programmes. One participant noted the importance of early exposure to these types of resources:

I think the most beneficial place for a video like this is in school for people going into primary or acute care. Because we never really had that discussion. In school, there wasn’t a class on ethics, or like how to give praise. There just wasn’t anything like that, so it was tough when you started (Participant 2, nurse practitioner, 6 years of experience).

Ongoing education was deemed essential by some participants to reinforce these principles, even for experienced health professionals. Recognizing the influential role of primary care clinicians in shaping clinic culture, participants highlighted the need for education to encompass all healthcare professionals and personnel in primary care settings: ‘If one person uses that language, it often translates to the rest of the staff’ (Participant 3, nurse practitioner, 0.5 year of experience).

Creating a supportive environment where stigmatizing language is replaced with stigma-free language and individuals on the healthcare team are supported, rather than punished, for their language use was considered crucial for fostering a culture of safety and respect. One participant stated:

Language is something you need to keep working on. Even if you know what is right or wrong, it takes time to build it into your brain and learn how to phrase what you think the right way. … We sometimes use inappropriate language, and we correct each other. So, I think it’s important to build a culture of safety and respect because we all make mistakes (Participant 14, physician, 8 years of experience).

The importance of considering patients’ perspectives was also highlighted, with one participant suggesting that observing how interactions affect patients ‘can hit home in a different way’ (Participant 1, nurse practitioner, 0.5 year of experience). Reflecting on their colleagues’ potential responses to the educational video, another participant acknowledged the prospect of learning even among ‘progressive’ healthcare practitioners.

I work with progressive providers who would say, … “We would never do that”. But there are always lessons in these things. I had a PA [physician assistant] student rotate through, and I thought this would be a great video for them to see. Depending on where you are and whether you’ve heard these messages before, it’s useful (Participant 11, physician, 1 year of experience).

Some participants emphasized the importance of examining personal biases and empowering patients in the context of T2D care. One participant noted:

I feel like the message is first checking our own biases, … and second, empowering patients to take control of a disease that is no fun. This definitely empowers them to make lifestyle modifications … and gives them the autonomy to do so without healthcare providers being super paternalistic and bossing them around. (Participant 5, nurse practitioner, 0.5 years of experience)

The necessity for a cohesive approach across all levels of healthcare delivery was underscored, with one participant emphasizing the systemic need for inclusive education and awareness to foster a culture of respectful and stigma-free communication about T2D within healthcare settings. ‘I think, in terms of language, the physician is just part of the medical team. Medical care starts from entering the door of the clinic, so the staff, the nurses, and the medical assistants should all use appropriate language’ (Participant 14, physician, 8 years of experience). Overall, it was recognized that the educational video’s impact and potential value within the primary care setting would depend mainly on context and prior exposure to these concepts, underscoring the need for such a tool across a broad audience.

## Discussion

This study examined the acceptability of an educational video designed to promote the use of stigma-free language among primary care clinicians when interacting with adults with T2D. Using a qualitative descriptive approach, the participants were interviewed about their views on messages in T2D care and their perceptions of the acceptability of the educational video. Analysis of the interviews revealed three primary themes: aligning video content with existing attitudes and beliefs, reducing the use of stigmatizing language, and increasing the use of stigma-free language. These themes offer valuable insights into enhancing the educational video and other teaching strategies, as well as designing future evaluations and applications.

Participants noted that the content of the video aligned with their pre-existing attitudes and beliefs regarding the crucial role of language in providing T2D care. They recognized that the language used in interactions with adults with T2D could influence care satisfaction and therapeutic relationships, highlighting a shared understanding of the benefits of using stigma-free language. Accounts emphasized the importance of language in promoting patient disclosure, building and maintaining trust, and supporting self-management. Additionally, the ethical duty to treat adults with T2D with respect and humanity emerged. Participants’ articulation of how the educational video aligns with their value systems implies that it fulfils key criteria for the acceptability of educational tools in healthcare. (Sekhon *et al.*, 2017)

Despite aligning with pre-existing attitudes and beliefs, participants also highlighted the challenges of changing ingrained habits and standard practices regarding the language used in patient-clinician interactions. Time constraints and heavy workloads were cited as potential barriers. Work-related burnout is prevalent among primary care clinicians, and emerging evidence suggests that clinicians who provide care for adults with T2D experience psychological distress, which can negatively impact the quality of their communication with adults with T2D. (Craven *et al.*, 2019; Beverly *et al.*, 2012) For some participants, the video highlighted the systemic issues within the primary care setting that hinder the use of stigma-free language, such as paternalistic attitudes and a negative framing of T2D. Such challenges underscore the need for approaches that account for the pressures and cultures in primary care practice, as well as routine reinforcement and institutional support. (van Brakel *et al.*, 2019)

Participants expressed a notable sense of optimism about the potential for continuous improvement in the language used within T2D primary care. Some highlighted the importance of health care professionals feeling empowered to engage in efforts to reduce stigmatizing language, encouraging an environment where they are not afraid to make mistakes. Many saw the educational video as a potentially valuable tool for new primary care clinicians and ongoing professional development. The emphasis on integrating this educational video into medical and nursing education curricula and periodic career development programmes was recurrent, suggesting an interest in sustained and structured learning environments. Some participants suggested that presenting more empirical evidence on the impact of language on clinical outcomes could enhance the credibility of the educational tool and foster greater acceptance among clinicians who are reluctant or traditionally minded. With the increasing access that health systems offer to health records, many health professionals, including primary care clinicians, are concerned that building and maintaining trust may be compromised when the language used evokes feelings of judgement and disrespect. (Fernandez *et al.*, 2021; Park *et al.*, 2021)

The educational video appeared to influence self-reflection, prompting some primary care clinicians to consider their language use in the context of T2D. This theme emphasizes the importance of practical and relatable educational tools that align with routine clinical experiences. Some primary care clinicians expressed their appreciation for the opportunity to identify with the characters portrayed in the educational video and valued the demonstrations of stigma-free language. The format of the educational video, short, accessible, and flexible, was well-received, indicating that viewing it could potentially be integrated into the busy schedules of primary care clinicians. (Sekhon *et al.*, 2017) Although education-based approaches have been examined to address stigma related to other chronic diseases, and the format of a brief educational video was viewed as acceptable, the video alone may not be sufficient to increase the use of stigma-free language by primary care clinicians in interactions with adults with T2D. One participant suggested adding an interactive component to a potential future intervention that includes the educational video. This idea is supported by research on addressing stigma related to chronic diseases, which suggests that combining interactive components with educational tools can lead to improvements in the use of stigma-free language. (Nyblade *et al.*, 2019)

Future research could quantitatively measure the impact of educational tools, as well as other approaches, on perceived and experienced diabetes-related stigma, patient satisfaction, diabetes self-management, psychological well-being, and glucose outcomes. (Dickinson *et al.*, 2024; Dickinson *et al.*, 2023) Furthermore, exploring the long-term effectiveness of language education and the persistence of behavioural changes among healthcare professionals could provide deeper insights into the sustainability of such educational tools and approaches. Implementing joint education sessions that include all healthcare team members, such as nurses, medical assistants, and administrative staff, could also be a promising approach to creating a unified and supportive clinic environment. (Nyblade *et al.*, 2019)

### Strengths and limitations

Study limitations include the use of a convenience sample, in which participating primary care clinicians volunteered to be interviewed. This may have resulted in the inclusion of individuals with a greater interest in clinician-patient communication in T2D care within the primary care setting. Differences in individual knowledge or skills related to stigma-free language use or diabetes management were not controlled for in this study, and such variability may have influenced the findings. Clinicians may have differed in baseline familiarity with ADA and ADCES guidance, previous training, or personal attitudes regarding language and stigma. Younger and less experienced clinicians, as well as female clinicians, might be more open to adopting stigma-free language and more likely to participate in this type of research, potentially leading to an overrepresentation of clinicians receptive to language-related practice change, while older or more experienced clinicians may be underrepresented. Additionally, physicians, nurse practitioners, and physician assistants may hold differing views on the use of language in T2D primary care. Broadly, the sample reflected national workforce data, with the primary care clinician workforce composed mostly of physicians and nurse practitioners in roughly equal proportions, and a smaller proportion of physician assistants. (HRSA National Center for Health Workforce Analysis, 2024) There were more primary care clinicians in the sample who identified as female than male. Ensuring equal participation of both female and male clinicians in future studies would contribute to a more comprehensive assessment of the educational video’s acceptability. The information obtained about the participants’ perceptions of the educational video may have been limited by the reliance on open-ended questions in the interview guide, which was designed to elicit perceptions from primary care clinicians. Future studies could incorporate pre-assessment of participant knowledge and skills or collect more detailed information on prior training and experience, to allow for a more nuanced interpretation of how differences in individual backgrounds may influence perceptions of acceptability.

## Conclusion

This study’s findings underscore the importance of increasing the use of stigma-free language in healthcare environments and provide data on how primary care clinicians might use and benefit from an educational video as a tool for promoting such changes. While study participants acknowledged existing challenges, their overall positive reception of the educational video and the self-reflection it inspired indicates an openness among primary care clinicians to receiving information about diabetes-related stigma and communication strategies to promote the use of stigma-free language in patient-provider interactions. Moving forward, integrating this formative research to improve the educational video and developing an expanded intervention that incorporates it holds promise for further enhancement and evaluation.
